# *Strongyloides stercoralis*: The Most Prevalent Parasitic Cause of Eosinophilia in Gilan Province, Northern Iran

**Published:** 2010-09

**Authors:** K Ashrafi, A Tahbaz, B Rahmati

**Affiliations:** 1 Dept. of Medical Microbiology, School of Medicine, Gilan University of Medical Sciences, Rasht, Iran; 2 Dept. of Infectious Diseases, Rasoul-Akram Hospital, Gilan University of Medical Sciences, Rasht, Iran

**Keywords:** *Strongyloides stercoralis*, Strongyloidiasis, Eosinophilia, Iran

## Abstract

**Background:**

Eosinophilia occurs in a wide variety of situations such as parasitic infections, allergic disorders, and malignancies. Most cases of eosinophilia of parasitic origin, especially those with a tissue migration life cycles consists of human infections by helminth parasites. The aim of present study was to determine the parasitic causes of eosinophilia in patients in a major endemic area of human fascioliasis in Gilan Province, northern part of Iran.

**Methods:**

One hundred and fifty patients presenting with an elevated eosinophilia attending infectious disease clinics with or without clinical symptoms, were examined. After clinical history evaluation and physical examination, coprological examinations were performed using the formalin-ether and the Kato-Katz techniques for detection of *Fasciola* sp. and intestinal parasites.

**Results:**

Forty two percent of patients were infected with *S. stercoralis,* nine (6%) were found to be infected with *Fasciola* sp. while only a single patient (0.7%) were infected by *Ttrichostrongylus* sp.

**Conclusion:**

Local clinicians in Gilan may consider eosinophilia as a suggestive indication for diagnosis of human fascioliasis, especially when microscopic stool and/or serological tests are negative. Based on the results, local physicians should consider *S. stercoralis* as the potential causes of eosinophilia in patients with elevated eosinophilia.

## Introduction

Eosinophilia is defined as an absolute count of >450/µl eosinophils in peripheral blood or as a WBC count in which more than 7% of WBCs are eosinophilic leukocytes, is mainly associated with a wide variety of infectious agents, especially helminthic parasites. The degree of eosinophilia in parasitic infections depends on the level of parasite contact with immune effector cells in host tissues. Therefore, eosinophilia is the highest among those parasites with a phase of development that involves migration through tissue (1). *Strongyloides stercoralis*, the causal agent of strongyloidiasis, is a soil-transmitted helminth commonly found in tropical and subtropical areas; infection rate is approximately 100 million people in about 70 countries all around the world. Strongyloidiasis usually manifest as a chronic intestinal infection with a spectrum of manifestations ranging from asymptomatic to life-threatening hyperinfection syndrome and disseminated disease (2–4). Although most of the human infections are asymptomatic and are diagnosed by accidental laboratory examination for other clinical situations, it will appear as a fetal disease in immunocompromised individuals and patients receiving corticosteroid chemotherapy (5–7). There is a strong correlation between strongyloidiasis and immunosuppressive disorders such as Human T-cell Lymphotrophic Virus-1 (HTLV-1) or Human Immunodeficiency Virus (HIV) and hematological malignancies (8–13).

The life cycle of *S. stercoralis* compromises of two free-living and parasitic cycles. The rhabditiform larvae (L_1_) pass in the stool will transform to adult free-living worms under favorable environmental conditions and establish the free-living cycle. These worms reproduce sexually and produce rhabditiform larvae in turn. In adverse environmental conditions the rhabditiform larvae prefer to become filariform ones (L_3_) and switch to parasitic cycle to infect humans. Autoinfection is a well-recognized feature in biology of *S. stercoralis*. During this important process the first stage larva (L1) transform to infectious filariform ones in small intestine after two molts and become adult worms following a lung migration (2, 14).

Strongyloidiasis is diagnosed by microscopy, agar plate culture and serological methods (15–21). However, agar plate culture technique is more effective and accurate than the other stool examination methods with a sensitivity of 78-100% (1, 18)-(19, 22). Combination of agar plate culture with formalin-ether concentration technique and agar plate with ELISA has been recommended to obtain better results (3, 18). Diagnostic sensitivity may reach 100% when seven consecutive stool samples are studied (23).

Peripheral eosinophilia is the most important laboratory finding seen in patients with strongyloidosis. Although eosinophilia could be considered as a good marker of *S. stercoralis* infection, it is not sufficiently sensitive to be used as a screening test for strongyloidosis (24–26).

Gilan Province, locating at the littoral regions of the Caspian Sea, has been an endemic area for a number of soil-transmitted helminths such as *Necator americanus*, *Ancylostoma duodenale*, *Trichuris trichiura*, *Ascaris lumbricoides* and *S. stercoralis* during last few decades. At present due to improvement of environmental conditions and hygienic standards, increasing the awareness of local residents about parasitic diseases, presence of better sewage disposal systems and availability of effective and safe anti-parasitic drugs, the prevalence of these parasites has decreased dramatically so that they are not considered as great public health problems, except for *S. stercoralis* (27)*.* The higher prevalence of the latter might be due to the complicate life cycle of the parasite, which includes a free-living one in addition to parasitic developmental stage.

In contrast, during last two decades a new public health problem, human fascioliasis, has emerged in Gilan province. Occurrence of two large outbreaks of human fascioliasis in the province in 1989 and 1999 has resulted in more than 15000 human infections (28–30). Hundreds of human cases have also been recorded annually between the outbreaks and thereafter. In most clinical cases, eosinophilia has been considered as a determinant factor for diagnosis and treatment of the disease, especially in those cases with negative stool microscopy.

Despite the drastic decrease in the prevalence of soil-transmitted helminthes in the province, *S. stercoralis* infection should be taken into consideration due to its dangerous nature in immunocompromised individuals and those cases receiving corticosteroid therapy. Unfortunately, the public health importance of strongyloidiasis is not fully considered in the province by physicians and many individuals with high eosinophilia have been treated as human fascioliasis.

The aim of the present study, however, was to clarify the parasitic causative agents of hypereosinophilic cases referred to our laboratory.

## Materials and Methods

During a five-year period, from February 2005 to February 2010, one hundred and fifty patients from different parts of Gilan province with the picture of hypereosinophilia were included in the current analysis. The patients were analyzed in a private clinic by a clinician specialized in infectious diseases. After evaluation of the clinical history and physical examination, patients were referred to the reference laboratory for more follow-up examination such as hematological, serological, and coprological examinations. In parallel, three fecal specimens of each patient were examined using formalin-ether concentration and Kato-Katz techniques. The fecal samples were examined on alternate days or during a period of 10 days. The formalin-ether method was used for detection of intestinal parasites, especially *S. stercoralis*, and Kato-Katz technique for *Fasciola* sp. All the sediments obtaining in formalin-ether method (at least eight 22×22 mm slides per sample per day) were examined under a light microscope using different magnitudes for detection of different parasites. The patients were under a liver-free diet during the study. All patients who were positive for different parasites treated under supervision of infectious disease specialist.

## Results

Of 150 subjects with high eosinophilia who referred to our laboratory, 88 (58.7%) were male and 62 (41.3%) were female. The patients were 14-78 (Average= 53.4, SD= 18.5) years old. The results show that 63 (42%) out of one hundred and fifty subjects were infected with *S. stercoralis* using formalin-ether concentration method from which 44 (69.8%) were male and 19 (30.2%) were female. Nine (6%) patients were infected with *Fasciola* sp. using formalin-ether and Kato-Katz techniques; 2 patients (22.2%) were male and 7 (77.8%) were female (Table 1). We also found one (0.7%) subject infected with *Trichostrongylus* sp. and four (2.7%) infected with *Giardia lamblia.* Mixed infection of *Fasciola* sp. and *S. stercoralis* was found in one case (0.7%), *S. stercoralis* with *Trichostrongylus* sp. in one case (0.7%), *S. stercoralis* and *G. lmablia* in 3 cases (2%), and *Fasciola* sp. with *Strongyloides* in one subject (0.7%). One patient (0.7%) was solely infected with *G. lamblia* only. Seventy-eight (52%) subjects had significant blood eosinophilia without any apparent parasitological cause after examination of three fecal specimens.

**Table 1:** Number and percentage of positive cases according to sex

Sex	Cases studied No. (%)	*S. Stercoralis* Positive (%)	*Fasciola* sp. Positive (%)	*Trichostrongylus* sp.Positive (%)	*G. lamblia* Positive (%)
**Male**	88 (58.7)	44 (69.8)	2 (22.2)	1 (100)	3 (75)
**Female**	62 (41.3)	19 (30.2)	7 (77.8)	–	1 (25)
**Total**	150 (100)	63 (100)	9 (100)	1 (100)	4 (100)

## Discussion

A wide variety of infectious agents, especially helminth parasites, are responsible for eosinophilia. Infections caused by protozoa (*Isospora belli*), fungi (*Coccidioides immitis* and *Aspergillus* sp.) and ectoparasites (*Sarcoptes scabiei*) to a lesser extent have also been associated with eosinophilia (1). Among helminth parasitic infections, strongyloidosis, fascioliasis, filariasis, trichinellosis, toxocariasis, and hook worms which undergo a tissue migration during their life cycles are reported to be more associated with persistent increase in the number of blood eosinophils (31, 32).

Parasitic infections due to soil-transmitted helminths are among the most prevalent causes of infection in humans. The majority of the infections are asymptomatic and severe infections are confined to the minorities of the general population. Microscopic examination of stool specimens, using different diagnostic techniques (direct wet smear, cup sedimentation, formalin-ether, flotation, and Kato-Katz methods) is used for diagnosis of most intestinal parasitic infections but it is not sensitive in mild and/or chronic infections. Based on this, multiple stool examination accompanied by hematological and serological tests might be warranted.

Many studies have evaluated the importance of eosinophilia in diagnosis of *S. stercoralis.* Loutfey MR et al. (33) showed that in 76 consecutive individuals with proven *S. stercoralis* infection, the majority (82.6%) of the patients had eosinophilia, suggesting eosinophilia as a potential useful marker for the diagnosis of the infection. Roman-Sanchez et al. (25) reported eosinophilia as the only indicator of *S. stercoralis* infection among farm workers on the Mediterranean Coast of Spain with a sensitivity of 93.5% and a specificity of 93.1%. Gill et al. (24) reported the eosinophilia as a highly suggestive marker of *Strongyloides* infections in world war II Far East prisoners. In another piece of study, Gill et al. (34) showed that the most important laboratory finding seen in patients with strongyloidiasis is eosinophilia. In spite of the importance of eosinophilia in diagnosis of the disease, all these studies have shown that the eosinophil count if used alone is not sufficiently sensitive to screen strongyloidiasis.

During last two decades, a significant decrease has occurred in prevalence and intensity of intestinal parasitic helminthes in Iran. Interestingly, despite the decrease in the prevalence of intestinal parasites in the country, another public health problem, fascioliasis, has emerged in Gilan province, northern part of Iran (27). Gilan province has long been considered as an important endemic region for hookworm infections, strongyloidiasis, ascariasis and trichuriasis. At present, the public health importance of these parasitic infections has significantly decreased in the province with prevalence of less than 1% in most areas (27, Ashrafi et al., unpublished data).

The great impact of fascioliasis on human populations and the potential risk of hyperinfection syndrome and disseminated strongyloidiasis in immunocompromised individuals and those who receive systemic corticosteroid therapy have resulted in high medical importance of these two parasitic infections in the province in spite of low prevalence of the infections.

The lack of a reliable serological test in the first large outbreak of human fascioliasis, occurred in Gilan province in 1989, made the clinician to diagnose the disease solely based on clinical history, stool exam and hematological characteristics, especially peripheral eosinophilia. After the outbreaks, the local physicians have considered eosinophilia as a key finding for diagnosis of human fascioliasis, and many suspected cases of the disease were treated each year only based on the presence of eosinophilia and nonspecific clinical manifestations. The results of our study showed that *S. stercoralis,* the most prevalent soil-transmitted helminth in the region, might be regarded as the most important parasitic agent of eosinophilia in clinical cases. Additionally, specific diagnostic techniques (agar plate culture and specific serological tests) must be used for exact determining of the causal agent of these cases.

The greater prevalence of strongyloidiasis in males (Table 1) could be related to their higher exposure to soil because of agricultural and gardening activities. About 94% of infected patients had a history of soil contact and 61% had agricultural activities as the main or second job. Due to some limitations, the authors were unable to use agar plate culture method for diagnosis of strongyloidiasis. As using of culture method improves the sensitivity of diagnosis, the number of real positive cases in our study could be higher than the ones that are reported here.Of nine cases who were infected by fascioliasis, two (22.2%) were male and seven (77.8%) were female. The role of the females in preparation of foods and salads and participation in agricultural activities in the area appears to have an important role in the higher prevalence of fascioliasis in this group (35, 36).

Chronic and asymptomatic nature of intestinal strongylodiasis and the potential risk for hyperinfection and life-threatening disseminated disease in immunocompromised patients warrants particular attention as to its diagnosis in everyone presenting with eosinophilia in the region.
